# A large-scale diffusion imaging study of tinnitus and hearing loss

**DOI:** 10.1038/s41598-021-02908-6

**Published:** 2021-12-03

**Authors:** Rafay A. Khan, Bradley P. Sutton, Yihsin Tai, Sara A. Schmidt, Somayeh Shahsavarani, Fatima T. Husain

**Affiliations:** 1 Beckman Institute for Advanced Science and Technology, University of Illinois at Urbana-Champaign, Urbana, IL 61801 USA; 2 Neuroscience Program, University of Illinois at Urbana-Champaign, Urbana, IL 61801 USA; 3 Department of Bioengineering, University of Illinois at Urbana-Champaign, Urbana, IL 61801 USA; 4 Department of Speech and Hearing Science, University of Illinois at Urbana-Champaign, Urbana, IL 61801 USA; 5 Department of Speech Pathology and Audiology, Ball State University, Muncie, IN 47303 USA; 6 Mortimer B. Zuckerman Mind Brain Behavior Institute, Columbia University, New York, NY 10027 USA

**Keywords:** Neuroscience, Auditory system, Cognitive neuroscience, Sensory processing, Anatomy

## Abstract

Subjective, chronic tinnitus, the perception of sound in the absence of an external source, commonly occurs with many comorbidities, making it a difficult condition to study. Hearing loss, often believed to be the driver for tinnitus, is perhaps one of the most significant comorbidities. In the present study, white matter correlates of tinnitus and hearing loss were examined. Diffusion imaging data were collected from 96 participants—43 with tinnitus and hearing loss (TIN_HL_), 17 with tinnitus and normal hearing thresholds (TIN_NH_), 17 controls with hearing loss (CON_HL_) and 19 controls with normal hearing (CON_NH_). Fractional anisotropy (FA), mean diffusivity and probabilistic tractography analyses were conducted on the diffusion imaging data. Analyses revealed differences in FA and structural connectivity specific to tinnitus, hearing loss, and both conditions when comorbid, suggesting the existence of tinnitus-specific neural networks. These findings also suggest that age plays an important role in neural plasticity, and thus may account for some of the variability of results in the literature. However, this effect is not seen in tractography results, where a sensitivity analysis revealed that age did not impact measures of network integration or segregation. Based on these results and previously reported findings, we propose an updated model of tinnitus, wherein the internal capsule and corpus callosum play important roles in the evaluation of, and neural plasticity in response to tinnitus.

## Introduction

Tinnitus, the perception of sound in the absence of an external source, is a highly heterogeneous condition in terms of its audiological features as well as its audiological, behavioral and psychological correlates. Tinnitus can be acute or chronic, and sufferers report tinnitus severity on a continuum from mild to severely bothersome. This is compounded by the fact that severity also changes with time; most sufferers tend to habituate to the sound, and their reported annoyance is seen to decrease with increased habituation^1^. This has historically made the condition difficult to study, as it has been difficult to account for the large amount of variation within the tinnitus population in terms of their tinnitus percept, time since onset, and severity, as well as associated comorbidities such as hearing loss. Tinnitus can have a highly debilitating impact on a sufferer’s daily life, and has been associated with increased levels of self-reported anxiety and depression, as well as diminished cognitive control^2^. To develop more effective therapies, it is necessary to develop a clearer understanding of the neural mechanisms underlying the percept of tinnitus.

The generation of tinnitus has been attributed to both changes in the periphery as well as changes in the central auditory pathway. While cochlear dysfunction is widely believed to hold a causal role in tinnitus^3^, we have yet to further elucidate the process of tinnitus onset and persistence. A leading theory accounting for the onset of tinnitus claims hearing loss can be a driver for tinnitus^4^. Tinnitus has a complex relationship with hearing loss; a majority of tinnitus sufferers have decreased hearing sensitivity, but only about half of those with clinically diagnosed hearing loss suffer from tinnitus^5^. Hearing loss is deprivation of stimulation to a system which expects stimulation (the auditory pathway), and tinnitus may be a result of compensatory mechanisms in response to the hearing loss. These mechanisms include increased spontaneous activity and/or reduced inhibition in the central auditory pathways^6^. However, this theory does not explain the approximately 20% of tinnitus sufferers with clinically normal hearing thresholds, or the 50% of those with hearing loss who do not develop tinnitus^5^.

Cognitive theories of tinnitus suggest attention plays a key role in the persistence of tinnitus^7–11^. In general, these models propose that frontal brain regions are involved in directing attention to the tinnitus signal, wherein sufferers are unable to ignore it. Another school of thought brings into consideration psychological as well as cognitive components. According to some theories^3,12,13^, tinnitus becomes chronic because frustration caused by the tinnitus percept leads to plasticity in auditory-limbic connectivity, leading to persistent tinnitus. While many of these theories are based on findings in neuroimaging studies, our lack of a clear understanding of the neural patterns underlying tinnitus has prevented the development of a holistic model of tinnitus. The identification of biomarkers of tinnitus could prove vital to our understanding and evaluation of these models, in addition to providing objective markers of tinnitus. The present study aimed to identify such biomarkers in white matter, while also investigating anatomical connectivity changes associated with tinnitus.

Investigations of white matter typically employ diffusion tensor imaging (DTI) methods to estimate microstructural integrity and orientation of white matter tracts in the brain, allowing for non-invasive observation of a wide range of features of these tracts. Fractional anisotropy (FA), which is a measure of the microstructural integrity of a fiber tract, is one of the most widely used DTI measures to study tinnitus. Mean diffusivity (MD), which represents general water diffusion in tissue regardless of direction, is also often studied in tinnitus. MD can also provide important context to FA results—while reduced FA is believed to underlie diminished microstructural integrity, confounding factors such as the presence of multiple fibers in a voxel can make conclusions less clear. If reduced FA is accompanied by an increase in MD, results are more likely to be driven by alteration in brain tissue^14^ (although DTI still lacks the necessary sensitivity to determine this with absolute certainty). Less commonly used in tinnitus research is fiber tractography, which allows us to identify specific tracts of interest and follow them along their full length, giving us detailed information about the various connections in the brain. Tractography also allows us to compute graph theory metrics on the DTI data, which can be used to model the connectivity between different regions of the brain^15^.

Investigations of white matter differences associated with tinnitus have thus far yielded a wide range of results, with many inconsistent findings (see Table 1). White matter plasticity in the auditory pathways has been reported in various studies^16–21^. Several studies also report alterations in limbic, and auditory-limbic connections in the brain^16,18,20–22^.

**Table 1:** Summary of findings from previous DTI studies of tinnitus.

Study	Subject groups	Major findings
Lee et al.^19^	28 TIN, 12 CON	Reduced FA in left frontal AF and right parietal AF for TIN group
Crippa et al.^16^	10 TIN, 15 CON	Stronger bilateral AC-AM connectivity, higher FA in AM-AC and AC-IC pathways, lower FA in IC-AM pathway in TIN group
Husain et al.^23^	8 TIN, 7 HL, 11 CON	Decrease in FA right corticospinal tract, SLF, ILF and ATR in HL as compared to CON. No difs in TIN group
Aldhafeeri et al.^17^	14 TIN, 14 CON	Reduced FA in right IF-OF, corpus callosum; left SLF, ILF and ATR in TIN compared to CON
Seydell-Greenwald et al.^21^	18 TIN, 14 CON	Increased FA in right AC and IC, left inferior IC. Significant correlation between tinnitus loudness and FA in bilateral vmPFC. Overall FA decreases with age and hearing loss
Benson et al.^40^	13 NIHL + TIN, 13 NIHL	Increased FA in left SLF, ATR, superior and anterior corona radiata, internal capsule; right SLF in NIHL + TIN group
Ryu et al.^20^	67 TIN, 39 CON	No FA differences between groups Decreased MD in superior, middle and inferior temporal WM, superior temporal sulcus, internal capsule, internal capsule, forinix stria terminalis, and sagittal stratum in TIN
Gunbey et al.^18^	18 TIN, 18 TIN_NH, 20 CON	Decreased FA in bilateral IC, MGB, TRN, AM, increase in FA for bilateral hippocampus for TIN as compared to CON. FA in LL decreased for TIN compared to both groups, and decreased in TIN_NH compared to CON
Schmidt et al.^24^	18 MLTIN, 19 BLTIN	No significant differences found
Chen et al.^22^	20 TIN, 22 CON	Reduced FA in genu of corpus callosum, left and right cingulum, and right superior longitudinal fasciculus; increased MD in the body of the corpus callosum for TIN compared to CON

*TIN* tinnitus group, *CON* normal-hearing controls, *HL* hearing loss controls, *NIHL* noise-induced hearing loss, *TIN_NH* tinnitus with normal hearing, *MLTIN* mild, long-term tinnitus, *BLTIN* bothersome long-term tinnitus, *FA* fractional anistropy, *MD* mean diffusivity, *AC* auditory cortex, *IC* inferior colliculus, *AM* amygdala, *AF* arcuate fasciculus, *IF-OF* inferior fronto-occipital fasciculus, *SLF* superior longitudinal fasciculus, *ILF* inferior longitudinal fasciculus, *ATR* anterior thalamic radiation, *vmPFC* ventromedial prefrontal cortex, *MGB* medial geniculate body, *TRN* thalamic reticular nucleus, *LL* lateral lemniscus.

Reflecting the heterogeneity of results in the literature, the superior longitudinal fasciculus^17,20,22^, inferior longitudinal fasciculus^17^, inferior fronto-occipital fasciculus^17^ corpus callosum^17,22^, hippocampus^18^, left arcuate fasciculus^19^ and right parietal arcuate fasciculus^19^ have also been reported to have varying degrees of plasticity across different studies In contrast, two studies^23,24^ found no white matter group differences between tinnitus subjects and controls. We have yet to identify consistent patterns in white matter metrics as they relate to tinnitus, which makes it challenging to understand the relationship between tinnitus and white matter integrity, if any exists. Some of this variance in the results may be driven by variables which have not been accounted for, such as hearing loss, age, or emotional disturbance. Overall, many studies enrolled small subject samples, leading to small effect sizes, and reporting large differences between findings across sites. The present study aimed to address some of the variability seen in the literature by enrolling a large cohort of participants, and also considering the independent and additive effects of hearing loss, to identify tinnitus-specific mechanisms and elucidate the relationship between tinnitus and hearing loss.

Studies investigating white matter plasticity in humans as it relates to hearing loss have revealed FA changes in the anterior thalamic radiation, inferior longitudinal fasciculus, and inferior fronto-occipital fasciculus^23^, and FA to be negatively correlated with hearing loss in white matter between left auditory cortex and corpus callosum^21^. A systematic review^25^ of 20 DTI studies of hearing loss found decreased FA measures were seen in a range of auditory-related brain regions, and that the auditory cortex and inferior colliculus were most widely reported to show diminished integrity in groups with hearing loss when compared to those with normal hearing.

The present study aimed to parse the relationship between tinnitus and hearing using DTI and tractography. To address some of the heterogeneity in the literature, a large sample of participants was recruited, while accounting for hearing loss and age. The aims were (1) to investigate FA and MD differences between tinnitus participants and controls, (2) to investigate FA and MD differences which could be independently attributed to hearing loss and tinnitus, as well as when they are comorbid, and (3) use tractography to calculate graph theoretical metrics between participant groups, which may implicate connectivity changes associated with tinnitus. Probabilistic tractography has been sparsely used in tinnitus research and could enrich our understanding of network-level anatomical changes associated with tinnitus. It was hypothesized that distinct anatomical neural networks relating to hearing loss and/or tinnitus could be identified using both FA and tractography. In order to reduce the impact that aging might independently have on the results, the tinnitus and control groups were age-matched—however, when participants were further divided into subgroups based on hearing acuity, there were some differences between them, and so age was included as a covariate of no interest in all contrasts.

## Results

### Participant data

There were no significant group differences in age (*t*(94) =  − 1.662, *p* = 0.1007), BDI-II scores (*t*(94) =  − 0.597, *p* = 0.552) or BAI scores (*t*(94) =  − 1.649, *p* = 0.103) between the CON and TIN groups.

When participants were separated into four groups based on tinnitus status as well as hearing acuity for the subgroup-level analysis, age significantly differed among the groups (*F*(3,92) = 12.19, *p* < 0.001). Post hoc analysis revealed that this significant finding was driven by the CON_HL_ group being older than the TIN_NH_ group (*t*(30.84) =  − 2.68, *p* < 0.05), by the TIN_HL_ group being older than the CON_NH_ group (*t*(27.76) = 4.490, *p* < 0.001), and TIN_NH_ group (*t*(20.93) = 4.395, *p* < 0.001).

BDI-II scores were not significantly different across the four subgroups (*F*(3,92) = 0.614, *p* = 0.608), but BAI scores were significantly different (*F*(3,92) = 2.974, *p* < 0.05). Post-hoc analysis revealed that this effect was being driven by a higher BAI score in the TIN_NH_ group as compared to the CON_HL_ group (*t*(18.91) = 3.261, *p* < 0.001). TFI scores were not significantly different between the two tinnitus subgroups (*t*(47.51) = 1.824, *p* = 0.07).

### FA analysis

#### Group-level analysis

**Figure 1:**
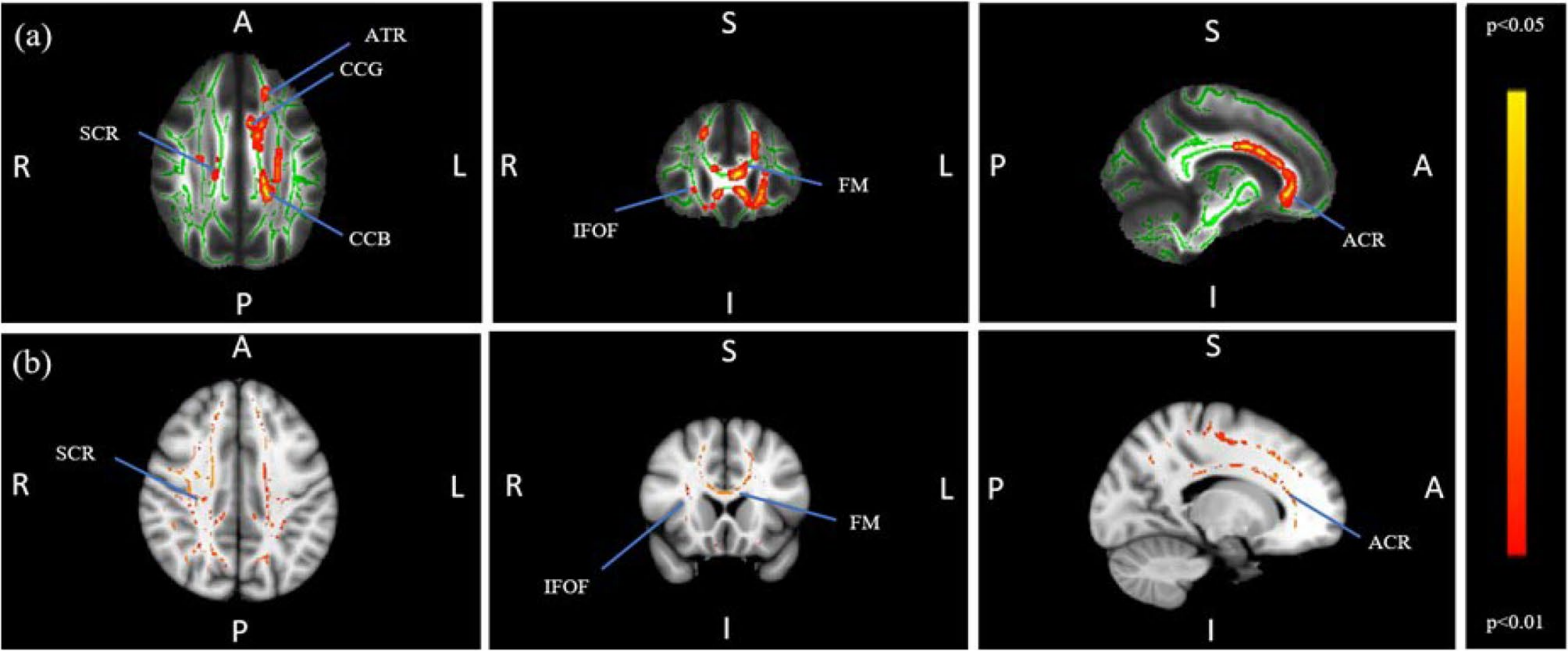
Row (**a**) Regions of significant difference in group level FA analysis (CON > TIN). Green represents the mean FA of all participants. The red-yellow scale represents regions of significant difference, with red representing regions of greatest difference. The regions showing significant differences between the groups included the right inferior fronto-occipital fasciculus (IFOF), right superior corona radiata (SCR), forceps minor (FM), genu (CCG) and body (CCB) of the corpus callosum, left anterior corona radiata (ACR), left anterior thalamic radiation (ATR), bilateral superior longitudinal fasciculus and left inferior longitudinal fasciculus (not visible in this view). Row (**b**) Regions of significant difference in group level MD analysis (CON < TIN). The red-yellow scale represents regions of significant difference, with red representing regions of greatest difference.

The CON < TIN contrast revealed no significant differences which survived threshold-free cluster enhancement (TFCE) correction for multiple comparisons. However, the CON > TIN contrast implicated several clusters encompassing regions of the right inferior fronto-occipital fasciculus, right superior corona radiata, forceps minor, bilateral superior longitudinal fasciculus, genu and body of the corpus callosum, left inferior longitudinal fasciculus, left anterior corona radiata, and left anterior thalamic radiation. Significant regions resulting from this contrast can be seen in Fig. 1.

MD analysis was conducted for any FA contrast seen to be significant. No significant group differences were seen for the CON > TIN contrast, but the TIN > CON contrast did demonstrate group differences, most notably in the superior corona radiata, forceps minor, inferior fronto-occipital fasciculus, and anterior corona radiata, reflecting a large amount of overlap with regions showing diminished FA in the TIN group (Fig. 1).

#### Subgroup-level analysis

**Figure 2:**
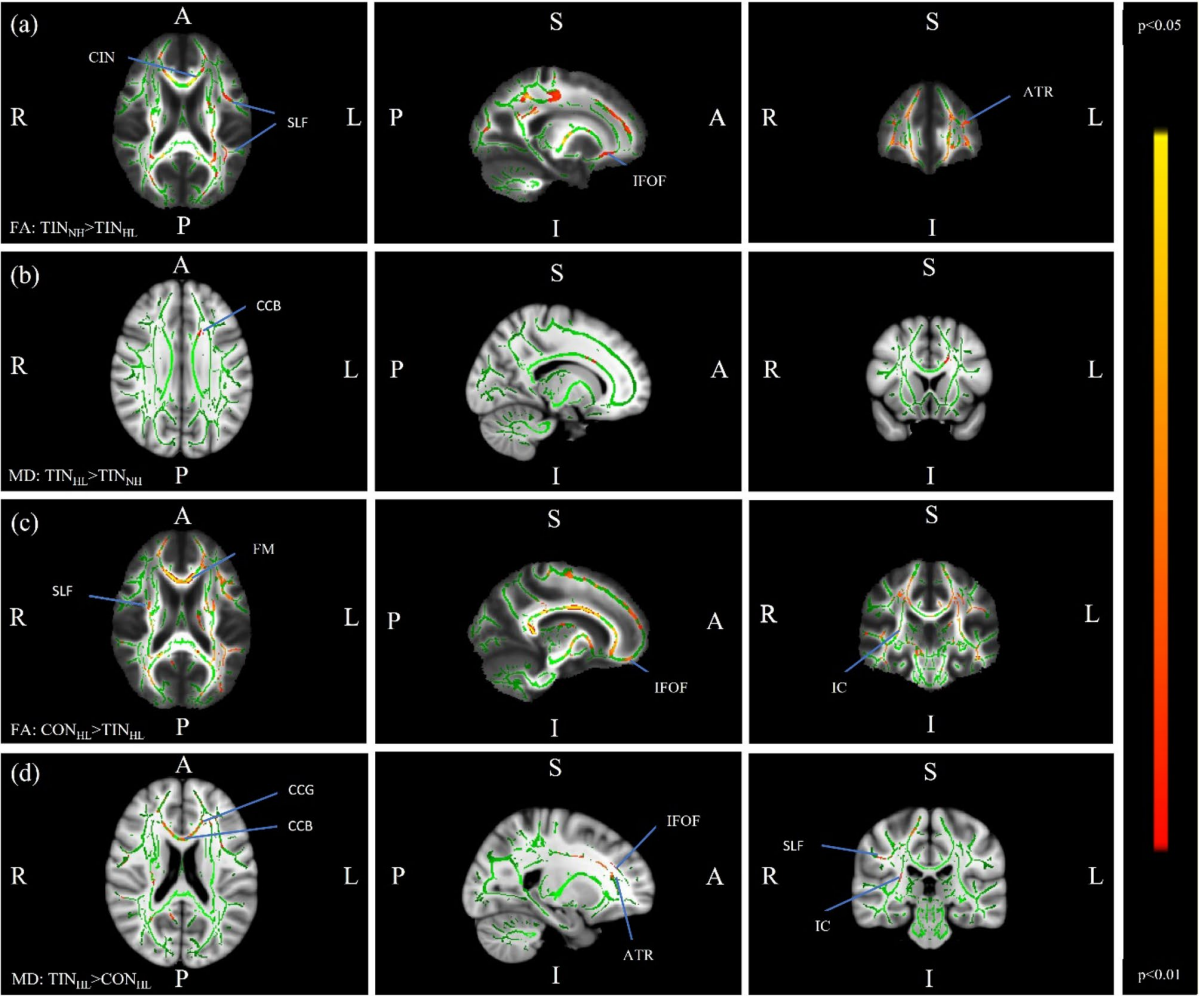
Regions of significant difference in subgroup-level FA and MD analyses (without age covariate). Green represents the mean FA of all participants. The red-yellow scale represents regions of significant difference, with red representing regions of greatest difference. Row (**a**) FA contrast for TIN_NH_ > TIN_HL_, where regions of significant difference include the left cingulum (CIN), left inferior-fronto occipital fasciculus (IFOF), left superior longitudinal fasciculus (SLF), left anterior thalamic radiation (ATR). Row (**b**) MD contrast for TIN_HL_ > TIN_NH_, where the only cluster that showed differences between the two groups was in the body of the corpus callosum (CCB). Row (**c**) FA contrast for CON_HL_ > TIN_HL_, where regions of significant difference include the forceps minor (FM), bilateral inferior fronto-occipital fasciculus (IFOF), right superior longitudinal fasciculus (SLF), and right internal capsule (IC). Row (**d**) MD contrast for TIN_HL_ > CON_HL_, highlighting similar regions of differences as row (**c**), in addition to the left anterior thalamic radiation (ATR).

To determine whether the results seen in the FA analysis were driven exclusively by the tinnitus status, or whether hearing acuity also played a role, subgroup FA analysis was conducted between the four participant subgroups divided based on tinnitus and hearing status. While there was a statistically significant difference in BAI scores between the TIN_NH_ and CON_HL_ groups, the mean scores for the two groups were 3.412 and 0.765 respectively (out of a total of 64 possible points on the scale). We did not expect such low scores to have an impact on results, so BAI scores were not considered for subscale analyses. An *F*-test revealed significant differences among the subgroups, and post-hoc *t*-tests illuminated the contrasts driving this result. Results from the subgroup FA post-hoc analysis are listed in Table 2. In summary, the only two contrasts that showed significant group differences were TIN_NH_ > TIN_HL_, and CON_HL_ > TIN_HL_. The left cingulum, left inferior-fronto occipital fasciculus, left superior longitudinal fasciculus and left anterior thalamic radiation showed decreased average FA values in the TIN_HL_ group compared to the TIN_NH_ group. The forceps minor, left inferior longitudinal fasciculus, right inferior fronto-occipital fasciculus, and right superior longitudinal fasciculus were seen to have decreased FA in the TIN_HL_ group as compared to the CON_HL_ group. However, when mean-centered age was added to this contrast as a covariate, no group differences survived corrections for multiple comparisons in either contrast. Figure 2 shows regions of significant differences in these contrasts.

**Table 2:** Subgroup contrasts for which FA analysis was conducted.

Contrast	Regions in significant clusters
CON_NH_ > CON_HL_	None
CON_NH_ < CON_HL_	None
TIN_NH_ > TIN_HL_	Left cingulum, left inferior-fronto occipital fasciculus, left superior longitudinal fasciculus, left anterior thalamic radiation
TIN_NH_ < TIN_HL_	None
CON_NH_ > TIN_NH_	None
CON_NH_ < TIN_NH_	None
CON_HL_ > TIN_HL_	Forceps minor, left inferior longitudinal fasciculus, right inferior fronto-occipital fasciculus, right superior longitudinal fasciculus, right internal capsule
CON_HL_ < TIN_HL_	None

Regions included in clusters that showed group differences in the contrast are indicated.

As in the group level analysis, MD analysis was conducted for significant contrasts at the sub-group level. The TIN_HL_ > TIN_NH_ contrast demonstrated a significant difference in the body of the corpus callosum, while the opposite contrast revealed no regions of significant difference. Similarly, the TIN_HL_ > CON_HL_ contrast showed differences in the genu and body of the corpus callosum, as well as regions of superior longitudinal fasciculus, internal capsule, inferior-fronto occipital fasciculus and anterior thalamic radiation, while the opposite contrast had no regions of significant difference. While the overlap of regions which showed reduced FA and increased MD was not as clear as in the group level analysis, the general pattern of results stayed the same (i.e. when a group was seen to have reduced FA, they were seen to have increased MD).

### Probabilistic tractography

Tractography metrics were calculated on the subgroup data. All three connectivity measures of interest were computed for four nodes—the left and right superior temporal lobes, and the left and right precuneus. Since the precuneus is a central structure, connectivity for it was computed as a single node by averaging the connectivity metrics between the two hemispheres within each participant, resulting in connectivity metrics from three nodes. Statistical analyses for the left and right superior temporal lobes were computed separately, due to the well-documented differences in the left and right Heschl’s gyri in auditory processing^26,27^.

Kruskal–Wallis tests revealed significant group differences in all three measures in the precuneus, mean strength at the right superior temporal lobe, and local efficiency and clustering coefficient at the left superior temporal lobe at an α value of 0.05. Post-hoc testing revealed that these significant results were driven by greater values for each measure in the CON_NH_ group compared to the TIN_HL_ group. In the precuneus, reductions in all three connectivity measures were seen for the TIN_HL_ group compared to the CON_NH_ group. In the right superior temporal lobe, the TIN_HL_ group had lower global mean strength compared to the CON_NH_ group, while the TIN_HL_ group also demonstrated reduced local efficiency and clustering coefficient in the left superior temporal lobe compared to the CON_NH_ group. Table 3 summarizes the findings from the tractography analyses.

**Table 3:** Results from Kruskal–Wallis significance tests for tractography metrics.

		Node (network represented by node)		
		Precuneus (DMN)	Right superior temporal lobe (AN)	Left superior temporal lobe (AN)
Mean strength	Result from Kruskal–Wallis test; effect size	*p*(χ^2^) = 0.0289; η^2^ = 0.0648	*p*(χ^2^) = 0.0175; η^2^ = 0.0767	*p*(χ^2^) = 0.0727; η^2^ = 0.0428
	Significant post-hoc contrasts	CON_NH_ > TIN_HL_*	CON_NH_ > TIN_HL_*	N/A
Local efficiency	Result from Kruskal–Wallis test; effect size	*p*(χ^2^) = 0.00754; η^2^ = 0.0963	*p*(χ^2^) = 0.0567; η^2^ = 0.0488	*p*(χ^2^) = 0.0282; η^2^ = 0.0654
	Significant post-hoc contrasts	CON_NH_ > TIN_HL_*	N/A	CON_NH_ > TIN_HL_*
Clustering coefficient	Result from Kruskal–Wallis test; effect size	*p*(χ^2^) = 0.00827; η^2^ = 0.0942	*p*(χ^2^) = 0.0361; η^2^ = 0.0596	*p*(χ^2^) = 0.0207; η^2^ = 0.0727
	Significant post-hoc contrasts	CON_NH_ > TIN_HL_*	N/A	CON_NH_ > TIN_HL_*

Post-hoc Dunn’s tests were conducted on significant contrasts, with Bonferroni correction. Effect sizes were calculated as η2 based on the H-estimate^66^. *DMN* default mode network, *AN* auditory network. *p < 0.05.

Given previous research that age may be associated with decreases in network integration and segregation, and because we saw significant group differences in age at the subgroup level, sensitivity analyses were conducted to account for potential effects of age in predicting each of the three tractography measures. Patterns of results were consistent after accounting for age, and age was not found to be associated with mean strength, local efficiency, or clustering coefficient at any of the three nodes.

## Discussion

The aims of this study were to evaluate the impact of tinnitus on neuroanatomy with and without hearing loss using diffusion imaging, to identify specific neural markers that could be used to distinguish subgroups based on their behavioral characteristics. We hypothesized that we would be able to detect distinct patterns of alterations to white matter architecture attributable to either tinnitus or hearing loss, or when both conditions were comorbid; this turned out to be true.

When participants were divided into subgroups based on hearing loss status, certain clusters showed reduced FA in the TIN_HL_ group compared to the TIN_NH_ group, and in the TIN_HL_ group compared to the CON_HL_ group (Table 2). However, these effects did not survive multiple comparison correction when mean-centered age was added as a regressor. This can be interpreted one of two ways—first it is possible that, as previously suggested, DTI findings in tinnitus can be explained by aging and extent of hearing loss^28^. Alternatively, because of the smaller subgroup samples (as in the CON_NH_, CON_HL_ and TIN_NH_ subgroups) there is insufficient power to overcome TFCE correction when age is added as a regressor of no interest, to parse out the comorbid effects of tinnitus and hearing loss^29^. Since the implicated regions in our results align closely with other studies reporting differences in white matter associated with tinnitus and hearing loss, and because we observed group-level differences in the CON versus TIN contrast when controlling for age, we assumed the latter for this discussion. However, we cannot rule out the possibility that much of the signal we are detecting can be attributed to age. For this reason, we were unable to identify distinct biomarkers for tinnitus and hearing loss while also accounting for age; however, such biomarkers may be identified with larger samples.

The organization of brain networks can be thought of in the context of graph theory, whereby certain highly connected nodes act as “hubs” of connectivity^30,31^. Crossley et al.^32^ found that numerous brain disorders were associated with disruptions in a few specific hubs, suggesting a relationship between the organization of neural architecture and such disorders. We believe tinnitus can be similarly understood. Specifically, we hypothesize that the specific network underlying tinnitus depends on the comorbidities which accompany it. Results partially supported a priori hypotheses—node-level group differences were seen in the precuneus, providing an analogue to findings in studies of functional connectivity^33–37^. Differences in connectivity were also seen in left and right superior temporal lobes, suggesting some reorganization of neural structure in auditory regions in the presence of both tinnitus and hearing loss.

Reduced FA was seen in the genu and body of the corpus callosum for TIN compared to CON, and in the forceps minor (a part of the corpus callosum) for TIN_HL_ compared to CON_HL_. Both contrasts also showed increased MD in these regions, with FA and MD findings collectively suggesting a reduction in microstructural integrity in the corpus callosum. The corpus callosum has previously been suggested as a possible driver for tinnitus persistence^22,38,39^. Reduced FA has been reported in the genu of the corpus callosum for tinnitus participants compared to controls^22^—a finding we were able to replicate in our group-level analysis. Further, the corona radiata and forceps minor also demonstrated differences in FA between the groups, echoing findings from Benson et al.^40^ and Aldhafeeri et al.^17^, respectively. It has been suggested that changes in interhemispheric connectivity in the corpus callosum may constitute a positive feedback loop between the primary drivers of tinnitus within each hemisphere, leading to tinnitus persistence^17,38,39^. Whereas our results replicate previous findings, the differences in hearing thresholds in both contrasts must be considered. Because the TIN and TIN_HL_ groups had worse hearing thresholds than the CON and CON_HL_ groups respectively, it is difficult to ascertain whether callosal plasticity is involved in the generation or persistence of tinnitus, or if it is a consequence of hearing loss.

**Figure 3:**
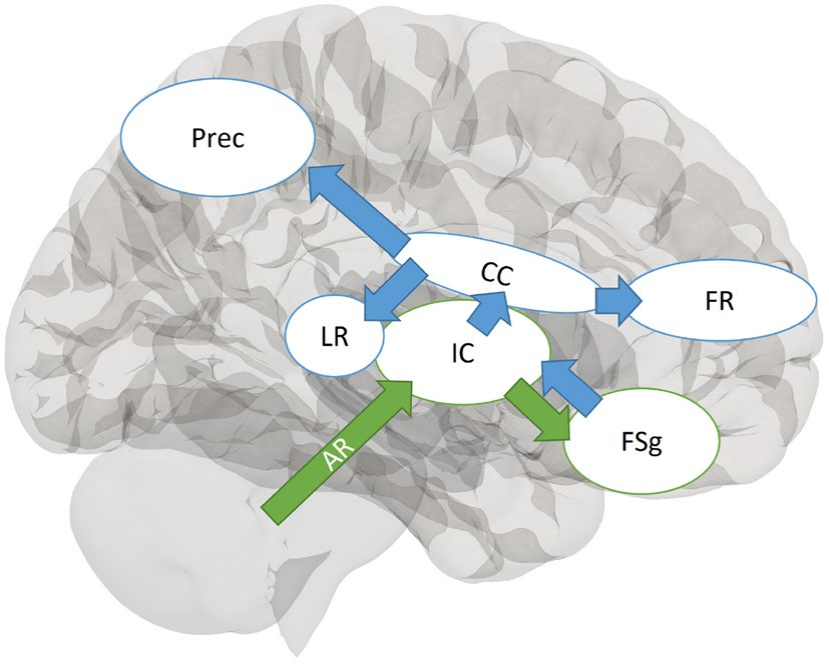
Diagram of proposed mechanism for tinnitus persistence. In this model, sensory signals from auditory radiations are propagated to the internal capsule, from where they are projected to the ventromedial prefrontal cortex and nucleus accumbens. There, frontostriatal gating as described by Rauschecker et al.^15^ takes place. Following evaluation of the tinnitus signal, frontal regions propagate signal back to the internal capsule, and the perception of a negative stimulus has a wider impact on limbic and frontal regions. Green arrows represent signal propogation prior to frontostriatal gating, while blue arrows represent the signal following frontostriatal gating. AR: acoustic radiations, IC: internal capsule, FSg: frontostriatal gating (consisting of the ventromedial prefrontal cortex and nucleus accumbens), CC: corpus callosum, FR: frontal regions, LR: limbic regions, Prec: precuneus.

The present study makes it apparent that when tinnitus is comorbid with hearing loss, associated neural plasticity can be differentiated from plasticity associated with tinnitus without hearing loss. Based on these observations, we propose a complementary paradigm for tinnitus to that put forth by Rauschecker et al.^13^, adding an emphasis on the role of the corpus callosum and internal capsule in the persistence of tinnitus (Fig. 3). Importantly, the only other study with a comparably large sample to the present study reported similar results to ours in the internal capsule and parts of the corpus callosum^40^. Rauschecker et al.^13^ proposed a frontostriatal gating system, in which the ventromedial prefrontal cortex and nucleus accumbens act as gatekeeping mechanisms for evaluation of sensory stimuli. The internal capsule, which was seen to have reduced FA in the TIN_HL_ group compared to the CON_HL_ group in the present study, is a waypoint for many ascending and descending fibers^41^, directly communicating with frontal regions of the brain. It is also a waypoint for auditory fibers, and is strongly connected with the corpus callosum. We propose that when an individual experiences auditory trauma, the internal capsule, which receives input from auditory fibers (such as the anterior thalamic radiation, which demonstrated reduced FA TIN_HL_ group compared to the TIN_NH_ group), acts as a waypoint and relays the signal to frontal regions, where frontostriatal gating takes place. This circuit then determines whether persistent tinnitus is onset. A frontostriatal circuit would consist of a closed-loop structure^13^, but the evaluation of a consistent negative stimulus would likely have widespread implications, which may include the altered, unbalanced callosal excitation and inhibition previously discussed^17,38^. Because the corpus callosum is one of the most widely connected neural structures, we believe that tinnitus-related alterations in other white matter structures (such as the superior longitudinal fasciculi, and interior fronto-occipital fasciculi) and brain networks (such as the default mode network) are likely a result of the persistence of tinnitus. These wider impacts are related to the cognitive and emotional aspects of tinnitus, as discussed earlier. In this model, changes in connectivity of the precuneus would likely be driven by constant awareness of the negative stimulus, as previously suggested by functional connectivity studies^42^. The precuneus plays an important role in the DMN, and the tinnitus signal would be a constant disturbance to the “rest” state, which may stimulate structural changes in the precuneus. Thus, the evaluation and persistence of tinnitus may occur at higher processing levels.

It is important to note that this proposed mechanism for tinnitus persistence is applicable only when tinnitus is comorbid with hearing loss. In the absence of hearing loss, it is unclear as to what kind of trauma or plasticity would induce the onset of tinnitus. Reduced FA in the cingulum for TIN_HL_ compared to TIN_NH_ may be a relevant finding. Previous studies have demonstrated tinnitus-related structural^22,24^ and functional^37,43–45^ plasticity in the cingulum, which plays an important role in inter-limbic signal propagation, but it is unclear why this would be more disrupted in one subgroup compared to others, given that measures of tinnitus severity were not significantly different between the groups. Future studies must address how tinnitus without hearing loss is different from tinnitus with hearing loss.

While the present study has numerous strengths over previous investigations, several caveats must be addressed. Like most other major neuroimaging studies of tinnitus, the investigation had a cross-sectional design, which makes it challenging to directly test our model. A series of longitudinal studies of tinnitus is required to answer many of our questions about the condition, which is currently a large gap of knowledge in the field. There are several limitations in our participant sample. Due to the nature of this study, participants were not randomly allocated to groups. We did not account for laterality of tinnitus or hearing loss in our analysis. It is possible that averaging across unilateral and bilateral tinnitus percepts and hearing loss erases smaller effects that may only be seen in specific conditions, but a larger focused study on laterality is required to test for this effect. In addition, there is a possibility that there may be many untested sources of confound which may influence our results. While hearing loss was not considered as a factor in the CON vs. TIN contrast, the average thresholds for the CON group were better than the TIN group in the 3000–12,500 Hz frequencies. Further, while differences between subgroups were statistically nonsignificant, the two normal hearing subgroups (CON_NH_ and TIN_NH_) appeared to have more closely matched thresholds than the two hearing loss subgroups (CON_HL_ and TIN_HL_), with the largest difference appearing between the 3000–6000 Hz range for the hearing loss subgroups. We are unsure as to why this pattern occurs, but because hearing loss can have an independent impact on FA results, any difference in hearing acuity may impact results. Finally, while age differences were accounted for by including age as a covariate of no interest in analyses, it is worth noting that the likelihood of both tinnitus and hearing loss increase with age. This means that, while it is important to regress out age due to its potential to confound results, we may possibly be losing some tinnitus or hearing loss-related effects in doing so. The effects seen in the subgroup FA analysis fell below the threshold for significance when age was added as a covariate, as noted earlier, which further complicates efforts to understand the exact role aging plays in these processes, although age was not seen to impact tractography results at the same subgroup level.

The study of tinnitus continues to be an imposing challenge to researchers in the field, and continued refinement of our models and theories is required as we aim to better understand this multi-faceted condition. The results reported here contribute significantly to the knowledge pool of tinnitus research, providing support for previously reported findings, while also reporting new findings which may prove important in our developing understanding. The proposed model integrates information from previous studies and updates our wider understanding of tinnitus, and we hope that further study will help us further refine our models and theories.

## Methods

### Study participants

A total of 96 participants were enrolled in this study. All participants provided informed consent as approved by the Institutional Review Board at the University of Illinois at Urbana-Champaign (#15955) and were suitably compensated. All procedures were conducted in accordance with the relevant guidelines and regulations. Participants were classified as having tinnitus if they self-reported having constant chronic tinnitus for at least the last six months before the study—all other participants were classified as controls.

Tinnitus severity was evaluated via the tinnitus functional index (TFI^46^). A 25 item scale, the TFI is widely used to assess tinnitus severity, and has been shown to have high internal consistency and reliability^47^. The main scale of the TFI is scored out of 100, and also consists of various subscales. Depression and anxiety were assessed via the Beck depression (BDI-II^48^) and Beck anxiety (BAI^49^) inventories, respectively. Both the BDI-II and BAI consist of 21 questions and can be scored out of a total of 63 points. For the present study, question 9 on the BDI-II (which assesses suicidality) was removed from the questionnaire. Both measures have been shown to be highly valid and reliable^50,51^. Participants with a history of traumatic brain injury, treatment for neurological diseases, Meniere’s disease, or BAI or BDI-II scores over 25 were excluded from participation.

**Figure 4:**
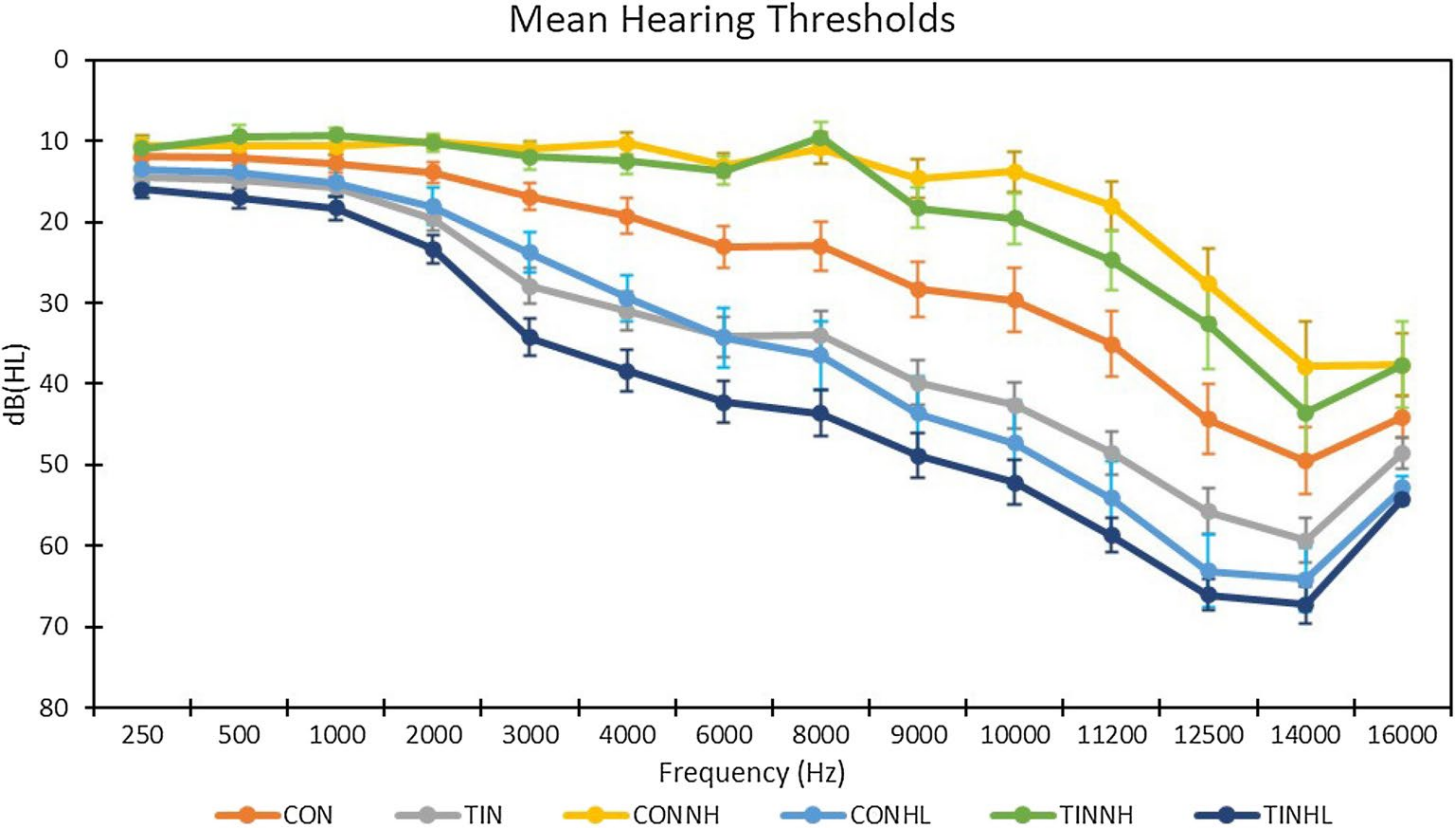
Bilateral average hearing thresholds for participants in each subject group. Bilateral hearing thresholds were averaged across ears within participant, and then across groups at each frequency. Error bars show standard error of the mean.

All participants underwent audiological testing. Pure tone thresholds were measured at 250, 500, 1000, 2000, 3000, 4000, 6000, 8000, 9000, 10,000, 11,200, 12,500, 14,000 and 16,000 Hz frequencies, and hearing loss was defined as hearing thresholds above 25 dB at any frequency between 250 and 8000 Hz in either ear. Hearing loss was considered asymmetric if there was a difference of 10 dB in thresholds between the two ears. Table 4 contains demographic data for all groups, while Fig. 4 contains bilateral mean hearing thresholds for each group.

**Table 4:** Mean ± Standard Deviation for participant demographics.

Group (n)	Age (years)	Gender	BAI	BDI-II	TFI score	Tinnitus laterality	Hearing loss laterality
CON (36)	47.78 ± 10.86	17 M, 19 F	1.55 ± 1.89	3.22 ± 5.14	N/A	N/A	N/A
CON_NH_ (19)	44.05 ± 10.11	7 M, 12 F	2.26 ± 2.25	4.05 ± 6.22	N/A	N/A	N/A
CON_HL_ (17)	51.94 ± 10.40	10 M, 7 F	0.76 ± 0.97	2.29 ± 3.55	N/A	N/A	B = 11, B(L) = 2, B(R) = 4
TIN (60)	51.65 ± 11.37	36 M, 24 F	2.38 ± 3.04	3.85 ± 4.72	23.40 ± 19.04	B = 50, L = 7, R = 3	N/A
TIN_NH_ (17)	41.29 ± 12.65	10 M, 7 F	3.41 ± 3.20	4.41 ± 5.81	17.58 ± 12.77	B = 14, L = 2, R = 1	N/A
TIN_HL_ (43)	55.74 ± 7.75	26 M, 17 F	1.98 ± 2.91	3.62 ± 4.27	25.70 ± 20.90	B = 36, L = 5, R = 2	B = 24, B(L) = 13, B(R) = 5, L = 1

*B* bilateral, *L* left ear, *R* right ear, *B(L)* bilateral, asymmetric left, *B(R)* bilateral, asymmetric right.

First, group-level FA analysis was run between all tinnitus participants (TIN, n = 60; mean age = 51.65 ± 11.37) and all non-tinnitus control participants (CON, n = 36; mean age = 47.78 ± 10.86), disregarding their hearing acuity. This was done to evaluate previous findings from our lab^23,24^ where no group differences due to tinnitus were seen. Then data were further divided into four subgroups based on their hearing and tinnitus status; non-tinnitus controls with normal hearing (CON_NH_, n = 19; mean age = 44.05 ± 10.11), controls with hearing loss (CON_HL_, n = 17; mean age = 51.94 ± 10.40), tinnitus subjects with normal hearing (TIN_NH_, n = 17; mean age = 41.29 ± 12.65) and tinnitus subjects with hearing loss (TIN_HL_, n = 43; mean age = 55.74 ± 7.75).

### MRI acquisition parameters

Data were acquired on a 3.0 T Siemens Prisma MRI scanner. High-resolution, T1-weighted, sagittal MPRAGE images were acquired with the following parameters: TR = 2300 ms, TE = 2.32 ms, flip angle = 8°, 192 slices, voxel size = 0.9 × 0.9 × 0.9 mm^3^. Diffusion image acquisition parameters were: TR = 8500 ms, TE = 81 ms, voxel size = 1.9 × 1.9 × 2.0 mm^3^, 72 slices, 60 diffusion directions, and a b-factor of 1000 s/mm^2^ with 2 b = 0 s/mm^2^ images acquired at the beginning of the run.

### DTI data analysis

Prior to DTI preprocessing and analysis, diffusion weighted data was preprocessed by converting DICOM files to NIFTI format using the dcm2nii tool (https://www.nitrc.org/projects/dcm2nii). DTI data analysis was completed the FMRIB Software library (FSL^52^ https://fsl.fmrib.ox.ac.uk/fsl/fslwiki). Eddy current correction was conducted using FSL’s *eddy* tool^53^, followed by skull stripping using the *BET brain extraction* tool. Reconstruction of diffusion tensors was then performed using the fMRI Diffusion Toolbox’s *DTIFIT* procedure^54^, which generated FA images for each individual participant.

Once diffusion tensors were calculated for all participants, group comparisons of FA values were conducted using tract based spatial statistics (TBSS) toolbox^55^. Each individual FA image was aligned to a 1 mm^3^ atlas space, and then mean a FA skeleton image was generated. Individual FA maps were projected onto the mean FA skeleton, and the *randomise*^56^ tool was used with 5000 iterations and threshold-free cluster enhancement (TFCE) to test for group differences. When evaluating group differences in terms of FA, only clusters with 100 or more contiguous voxels were considered. For the group-level FA analysis, a contrast was run between the TIN and CON groups, with mean-centered age included as a regressor of no interest. For the subgroup-level analyses, an *F-*test was conducted between the CON_NH_, CON_HL_, TIN_NH_, and TIN_HL_ groups.

To supplement the FA analysis, MD was evaluated for the contrasts that showed significant FA differences. While FA is often used to represent microstructural integrity of a tract, there are other confounding factors which may impact the measure. MD is a measure which does not account for direction of water diffusion, and thus provides important contextual information to FA results. MD analysis was conducted in a similar fashion to FA analysis—individual MD images were aligned to a 1mm^3^ atlas space using the same transformation parameters as individual FA images. Following image transformation, TBSS was conducted using the *randomise*^56^ tool, as in FA analysis.

### Probabilistic tractography

Fiber tracking was conducted in a manner similar to that reported by Sharp et al.^57^. FSL’s *bedpostx* tool^54,58^ was used to estimate the probability distribution of fiber orientations at a voxel-level. Cortical parcellation was conducted on individual T1-weighted anatomical images using Freesurfer’s *recon-all* tool^59^, generating 82 regions of interest—68 cortical regions and 14 subcortical regions, which made up the seed/target regions of the connectome. Each region was then registered to the subject’s DTI space using the Freesurfer *bbregister* tool^60^, with FSL’s *FLIRT*^61,62^ used to calculate the transformation matrix of the images from diffusion-weighted space to T1-weighted space. The inverse of this matrix was then used with Freesurfer’s *mri_vol2vol* to move the Freesurfer parcellation into diffusion-weighted space.

Following this process, probabilistic tractography was conducted. 5000 seed streamlines were generated from each voxel within each of the parcellated regions. Targets were defined as any voxel within any of the 81 target regions. FSL’s *probtrackx2*^58^ was used to generate a matrix which contained the number of streamlines from each seed volume that reached all other 81 target regions. The processing was also set up to avoid ventricles. Following this, each of the 82 entries in the connectome was normalized by the average volume of both ROI’s in the pathway. The weighted connectomes were then symmetrized by averaging identical entries that initiated from opposite ends of the tract. Finally, each connectivity pair in individual matrices were averaged within subgroups, resulting in a mean connectivity matrix for each subgroup.

The brain connectivity toolbox (BCT)^63^ was then used to compute graph-theoretical analyses on the symmetrized, weighted connectomes. Based on findings in functional connectivity literature, connectivity was examined at three nodes—the precuneus and the left and right superior temporal lobes. The precuneus was selected because it is an important node in the default mode network, while the superior temporal lobe is the location of the primary auditory cortex and is involved in the auditory network. Both networks have been shown to have altered activity in tinnitus subjects^42^. Three graph-theoretical metrics were examined for each of the nodes of interest—mean strength, local efficiency and clustering coefficient. Mean strength averages the connection strength across all nodes in the connectome, and so represents global structural connectivity. Efficiency is a measure of how efficiently information is exchanged in a network, and local efficiency refers to the application of this measure within smaller subnetworks. This is calculated as the average inverse topological distance between nodes. Finally, the clustering coefficient is a measure of how well clustered together nodes in a graph are. Mean strength and local efficiency are indicators of network integration, while clustering coefficient is an indicator of network segregation. These network measures were selected to give us an understanding of possible alterations in network architecture which may be associated with tinnitus. Statistical analysis on the metrics acquired from BCT was conducted using the R programming language (R Core Team version 3.6.1, 2019)^64^. Since some of the participant subgroups had relatively small sample sizes, non-parametric Kruskal–Wallis tests were conducted to evaluate group differences in each of the three measures in each node. For each contrast demonstrating significant effects through Kruskal–Wallis tests, Dunn’s test was conducted to identify the contrast driving the significance, corrected using Bonferroni correction. Effect sizes for all analyses were conducted using the *rstatix*^65^ package in R. These metrics were originally evaluated parametrically using ANOVA, Tukey’s HSD and false discovery rate (FDR) correction, but upon suggestion from a reviewer, were reported via non-parametric tests. Differences between the analyses were marginal—results from ANOVA and Kruskal–Wallis were nearly identical in their findings, with only two post-hoc tests which were significant in parametric analyses showing non-significant *p* values in parametric results.
